# Corpus Callosotomy for Controlling Epileptic Spasms: A Proposal for Surgical Selection

**DOI:** 10.3390/brainsci11121601

**Published:** 2021-12-01

**Authors:** Tohru Okanishi, Ayataka Fujimoto

**Affiliations:** 1 Division of Child Neurology, Brain and Neurosciences, Faculty of Medicine, Tottori University, Yonago 683-8503, Japan; 2 Comprehensive Epilepsy Center, Seirei Hamamatsu General Hospital, Hamamatsu 430-8558, Japan; afujimoto.scienceacademy@gmail.com

**Keywords:** corpus callosotomy, epilepsy surgery, epileptic spasms, West syndrome, adaptation, surgical selection

## Abstract

In 1940, van Wagenen and Herren first proposed the corpus callosotomy (CC) as a surgical procedure for epilepsy. CC has been mainly used to treat drop attacks, which are classified as generalized tonic or atonic seizures. Epileptic spasms (ESs) are a type of epileptic seizure characterized as brief muscle contractions with ictal polyphasic slow waves on an electroencephalogram and a main feature of West syndrome. Resection surgeries, including frontal/posterior disconnections and hemispherotomy, have been established for the treatment of medically intractable ES in patients with unilaterally localized epileptogenic regions. However, CC has also been adopted for ES treatment, with studies involving CC to treat ES having increased since 2010. In those studies, patients without lesions observed on magnetic resonance imaging or equally bilateral lesions predominated, in contrast to studies on resection surgeries. Here, we present a review of relevant literature concerning CC and relevant adaptations. We discuss history and adaptations of CC, and patient selection for epilepsy surgeries due to medically intractable ES, and compared resection surgeries with CC. We propose a surgical selection flow involving resection surgery or CC as first-line treatment for patients with ES who have been assessed as suitable candidates for surgery.

## 1. Introduction

In 1940, van Wagenen and Herren [1] first introduced the corpus callosotomy (CC) as a surgical treatment option for patients with medically intractable epilepsy and reported the efficacy of preventing the propagation of focal seizures to the contralateral hemisphere. Since then, CC has been applied for the treatment of drop attacks, which were classified into generalized tonic or atonic seizures in the latest classification of seizure types in 2017 by the International League Against Epilepsy (ILAE). However, CC has also been adopted for the treatment of epileptic spasms (ES), as reported by Pinard in 1993 [2], and reports concerning CC for the treatment of patients with ES has increased since 2010 [3,4,5,6,7,8,9,10,11,12].

ES are epileptic seizures characterized as brief muscle contractions that typically involve the axial muscles, proximal limb segments, and a main feature of West syndrome [13]. It has been hypothesized that the epileptogenic cortex triggers the activation of subcortical structures, leading to ES [14,15]. Resection surgeries, including cortical resections, frontal/posterior disconnection and subtotal/total hemispherotomy/-rectomy, have been established for the treatment of ES in patients with epileptogenic regions estimated to be located in the unilateral hemisphere [16,17,18,19,20]. However, CC has also been a helpful treatment option for patients without apparent lateralized epileptogenic findings on presurgical evaluations.

Here, we review relevant literature concerning CC and related adaptations, and discuss pertinent physiology, ES, and epilepsy surgeries. There have been no studies discussing the surgical selection of resection surgery or CC in patients with medically intractable ES. We propose a selection flow of epilepsy surgeries for medically intractable ES, including resection surgery and CC.

## 2. History of Corpus Callosotomy, Types of Disconnections and Complications

Van Wagenen and Herren encountered three patients with refractory epilepsy who had decreased seizure frequency or bilateral spreading due to tumors or hemorrhage involving the corpus callosum. They undertook disconnection of the corpus callosum in those patients with medically intractable epilepsy, and reported the efficacy of preventing propagation of focal seizures to the contralateral hemisphere (secondary generalization) and developing generalized seizures or loss of consciousness [1]. In the mid-twentieth century, this procedure received little attention. In 1962, Bogen and Vogel reported a patient who had undergone a total (complete) CC, who experienced intractable seizures due to a head injury sustained during World War II, and CC became a surgical treatment option for intractable seizures [21,22].

Compared with total CC, acute and chronic disconnection syndromes have been found to be less and milder in patients where CC surgery spared the posterior portion of the corpus callosum [23]. Fiber tract studies using magnetic resonance imaging (MRI) have shown that the posterior corpus callosum transfers perceptual information [24]. An anterior 1/2 to 4/5 CC technique has been selected for patients with intractable epilepsy to reduce disconnection syndromes [22,24,25]. For patients who demonstrate meaningful language, the anterior CC technique has been proposed as a first surgical treatment option [25]. When anterior CC has been found to be ineffective or insufficient, additional disconnection of the rest of the posterior corpus callosum has been performed (two-staged CC). Conversely, disconnection syndrome is absent or mild before 10 years of age even after total CC [26]. In patients under 10 years of age with severe cognitive impairment or unilateral widespread cerebral lesions, a total CC has been selected as the initial step for reducing surgical complications. Two recent studies have reported the use of a posterior 1/2 CC technique, involving disconnection in the splenium, isthmus, and posterior of the body in the corpus callosum, to treat drop attacks [27,28]. These studies showed that the efficacy of posterior CC corresponded to a total CC for drop attacks with fewer disconnection symptoms.

Other than disconnection syndrome, the surgical complications in CC are rare (3%) comparing with general data of epilepsy surgery (11%) [29]. CC is considered as relatively safe epilepsy surgery.

## 3. Mechanism of Efficacy and Adaptations to the Corpus Callosotomy

The corpus callosum is the largest interhemispheric commissural structure in humans. Almost 70% of cerebral cortices are connected by the corpus callosum [24,30]. The physiological role of the corpus callosum is to equalize and balance bilateral cerebral activities in normal status [24]. In an epileptic brain, focal seizures propagate to the contralateral hemispheres, resulting in secondary generalization mainly via the corpus callosum. The anterior portion of the corpus callosum plays an essential role in the generalization of tonic and tonic–clonic seizures [24]. In one analysis of patients with ES, a part of the ES was generated through interhemispheric recruitment of bilateral epileptic activities via the corpus callosum [31].

The CC procedure has been considered as palliative epilepsy surgery which means that the goal is not for patients to be seizure-free. Adaptations to the CC procedure have been applied to patients with intractable seizures, to patients who were not suitable candidates for cortical resection, and to patients whose main seizure type is a ‘drop attack’ [22,25,28,32,33,34,35,36]. Patients with rapid secondary generalization of focal seizures are also suitable candidates for CC. Patients with generalized onset seizures of atypical absence and generalized tonic–clonic seizures can also be suitable candidates for CC, although it is less efficacious in such patients than in those with drop attacks [32,37,38,39]. Like epilepsy syndrome, Lennox-Gastaut syndrome and similar forms of epilepsy syndrome (Lennox-like syndrome), which have been described as symptomatic generalized epilepsy in the 1989 ILAE epilepsy classification, have been the adaptation of CC [40].

Although the term ‘drop attack’ has typically been used for patients whose seizures manifest as a sudden fall, this term was not defined in the latest ILAE seizure classification in 2017 [41], whereas multiple seizure types were defined. Few studies have specifically defined drop attacks. Ikeno and Fukushima et al. investigated drop attacks, defined as a sudden falling down due to the seizure itself, and they reviewed video-electroencephalography (EEG) records of seizures meeting this definition [42,43]. They concluded that drop attacks included generalized tonic seizures, atonic seizures, flexor spasms, and myoclonic atonic seizures [42,43]. In contrast, in previous studies concerning CC, the term ‘drop attacks’ has referred to generalized tonic and atonic seizures [5,24,25,35,36,38,44]. Based on these previous studies, drop attacks are presumed to be generalized tonic and atonic seizures that develop, in the latest seizure ILAE classification.

## 4. Epileptic Spasms: Characteristics of Clinical and Neurophysiological Findings, Mechanisms, and Treatment Options

Previous studies have identified the following three contiguous patterns of ictal EEG findings during ES: (i) fast waves or high-frequency oscillations preceding the ES, (ii) high-voltage slow waves (HVSs), and (iii) desynchronization [14,45]. Among these, HVSs coincide with the phasic motor component of ES and occur in 100% of cases [13,45].

Although ES is one representative manifestation of West syndrome and usually occurs in the first year of life [13,14], it can occur in patients up to 14 years old with intractable epilepsy and epilepsy syndromes such as Lennox-like syndrome, epileptic spasms without hypsarrhythmia, Lennox-Gastaut syndrome, epilepsy with myoclonic–atonic seizures, and Dravet syndrome [46,47,48,49]. The neurophysiological mechanisms underlying ES remain largely unknown. Previous studies have shown that both the cortex and subcortical structures, including the subcortical white matter, brainstem, thalamus, and basal ganglia, play a role in the emergence of ES [15,50]. Researchers have hypothesized that ES are derived from cortical–subcortical interactions wherein the cortex triggers activation of subcortical structures, thereby leading to the generation of ES [14,15] and several neurophysiological and pathological studies revealed the phenomenon supporting the hypothesis. The cortical epileptic excitations are stronger and more widespread on the cerebrum presenting with ES than those without ES [51]. The oligodendroglia-like cells distribute extensively on gray-white matter junction and white matter in the patients representing ES [52]. The analysis for spatial concordance between fast and slow EEG activity indicated subcortical contribution in the emergence of ES [53]. Interhemispheric modulation and recruitment of epileptic excitations via the corpus callosum are necessary for the emergence of at least some part of the ES [10,11,12]. The etiology is multiple and diverse and has been classified as structural, genetic, infectious, metabolic, immunological, or involving unknown conditions [54]. ESs are considered a common neurophysiological manifestation in different conditions with extended cortical and subcortical epileptic excitations in children.

Adrenocorticotrophic hormone (ACTH) and oral corticosteroids have been established as the most effective treatments for ES in infants and are reported to control seizures in 75% of these patients [54]. Vigabatrin has shown less efficacy in all patients than ACTH; however, it has better efficacy in ES in patients with tuberous sclerosis complex compared with hormonal treatments [54]. Other treatment options involving antiepileptic drugs, dietary therapy, and epilepsy surgery have been selected for patients whose first-line treatment was unsuccessful. Vagus nerve stimulation is less effective for medically intractable ES [26,55]. Hormonal treatments have also been selected to treat late-onset ES, although few studies have investigated these treatments [46,56].

The early treatment from the onset and the treatment success are associated with favorable seizure and cognitive outcomes in the patients with ES. In cryptogenic West syndrome, which is supposed to be classified into unknown or genetic etiology in the latest classification of ILAE, the start of ACTH therapy within one month from the onset of ES is recommended [57,58,59]. If the treatments are insufficient, the patients with West syndrome often evolve into Lennox-Gastaut syndrome or Lennox-like syndrome which develops quite intractable seizures [54]. The duration of these epileptic encephalopathies is longer, the development of the patients is more disrupted. The best possible treatment options should be tried during the infantile or early childhood period.

## 5. Corpus Callosotomy for Epileptic Spasms

Prior to 2000, there were few reports concerning the use of CC to treat patients with ES [2,60,61]. Talwar et al. reported three patients aged between 5 and 12 years who had undergone CC surgery, with two of the patients having experienced a marked reduction in ES frequency [60]. In 1999, Pinard et al. reported a case series involving the use of CC to treat patients with ES [61] involving ten patients (age range, 2–9 years) with symptomatic generalized epilepsy presenting with ES, who received one or two stages of total CC. In that case series, CC was found to be insufficiently effective; eight patients developed tonic seizures, with each patient showing asymmetrical spasms and head nodding postoperatively. Between 2000 and 2009, a limited number of studies concerning CC, including some patients with West syndrome, have been reported [62,63]; however, its efficacy in treating patients with ES or West syndrome was not elucidated.

Since 2010, the number of studies concerning the use of CC to treat patients with ES has increased. Some studies have not provided particular details concerning the efficacy of CC procedures to treat ES or West syndrome, but have reported whole outcomes including patients with other types of seizures, Lennox-like syndrome, or Lennox-Gastaut syndrome [64,65,66,67,68,69]. However, in other recent studies, the outcomes of CC to treat ES, which mostly involved patients with West syndrome, have been presented using detailed patient data [3,4,6,8,9,10,11,70]. Because of these studies, CC use has been adapted to not only involve treatment for drop attacks (tonic seizures, atonic seizures) and atypical absence and tonic–clonic seizures, but also for patients with ES, and for older patients with Lennox-Gasutaut syndrome and Lennox-like syndrome, as well as infants with West syndrome, regardless of the presence of the sudden falling-down manifestation.

## 6. Efficacy of Corpus Callosotomy for Epileptic Spasms

### 6.1. Seizure Outcomes Post-Corpus Callosotomy for Epileptic Spasms

Although most previous studies regarding the efficacy of CC for drop attacks have not provided data on efficacy, particularly for ES, several studies have described CC outcomes in relation to ES [3,4,6,8,9,10,11,12,61,70] as shown in Table 1. Seizure-free ratios post-CC ranged from 25% to 79% among the studies. In a relatively large study, Otsuki et al. reported that CC achieved seizure freedom in 7 of 15 patients (54%) with West syndrome and in 7 of 10 (70%) patients with ES [4]. Baba et al. [6] reported the efficacy of CC in 56 patients (age range, 5.1–22.6 months old at CC; mean follow-up, 36.6 months) with intractable West syndrome without brain lesions on MRI. Regarding patients with ES, they reported that 43% of these patients who underwent CC achieved seizure freedom, 23% achieved ≥80% seizure reduction, and 13% achieved ≥ 50% seizure reduction at the last follow-up, whereas in patients with tonic seizures, no seizure freedom was observed [6]. Kanai et al. also reported 17 patients (age range, 17 months–19 years; mean follow-up, 22.1 months) with West syndrome (*n* = 10 patients) or Lennox-like syndrome (*n* = 7 patients) who received CC for the treatment of ES [10]. In that study, they evaluated and selected patients with ES that strictly met the definitions of ictal EEG [13,45] using video-EEG monitoring. They reported that 41% of patients who underwent CC achieved seizure freedom (Engel classification I). In a report with a small number of patients, Iwasaki et al. [70]. reported eight patients with ES who had been treated with a total CC, of whom three patients achieved seizure freedom. Okanishi et al. reported seven patients with bilateral multiple cortical tubers in the tuberous sclerosis complex who underwent a CC for ES. Of these, three patients achieved seizure freedom with CC only, and two achieved seizure freedom through additional treatments [9]. In the patients with partial response in those previous studies, CC achieved reduction of seizure frequency and/or changed the ES into smaller ones, like head nodding or resolving drop attacks, unilaterally focal onset ES, or bilaterally independent focal onset-ES [3,4,6,8,9,10,11,12,61,71].

Large data of long-term outcomes of CC for ES is unavailable. Stigsdotter-Broman reported the data of long-term outcomes of CC for tonic seizures and atonic seizures, and, in this study, the total seizure frequency gradually decreased over 5–10 years of follow-up [72]. The efficacy of CC preventing interhemispheric recruitment of bilateral epileptic activities may contribute to suppression of epileptic aggravation. There also has been no evidence that the CC prevents the evolution from West syndrome to Lennox-Gastaut syndrome. However, the seizure outcome is poor in the patients who have not only spasms but also tonic seizures [6] and cases that have evolved from West syndrome into Lennox-Gastaut syndrome [73]. CC within the period of ES only or West syndrome might be warranted in the patients with unsuccessful medical treatments.

Occasionally, CC fails to achieve seizure freedom and ES continue from the unilateral hemisphere as an asymmetrical feature. In this situation, additional resection surgeries, such as cortical resection, anterior or posterior disconnection, or total or subtotal hemispherotomy/hemispherectomy are performed after CC. In previous studies, seizure-free ratios, including patients who received CC plus additional resection surgeries, ranged from 43% to 71% [3,7,8,9,12]. The duration from CC to additional resection surgeries have become shorter in recent studies (1–6 months) [9,12] than previously reported (6–62 months) [3]. The efficacy of CC usually become apparent soon after the procedure. For the better seizure and cognitive outcomes, the early evaluation might be recommended in residual ES cases after CC.

### 6.2. Effects on Developments in Patients with Epileptic Spasms

Some studies have reported the effects of CC on patient development. In one study concerning the use of CC to treat patients with West syndrome, patients with a successful outcome (≥80% seizure reduction) had better developmental outcomes than those with poorer outcomes (<80% seizure reduction) at one and two years postoperatively [6]. Honda et al. investigated changes in the development of 106 patients who had undergone CC surgery prior to age 6. Among 89 patients who presented with ES, 44% were reported to have improved developmental quotients one year postoperatively. In data concerning 106 patients, low preoperative developmental quotients and postoperative seizure freedom were found to correlate with developmental improvements due to CC surgery [66].

## 7. Prognostic Factors for Seizure Outcomes Post-Corpus Callosotomy

In a study of CC non-specific to ES but including other types of drop attacks, total CC (compared with anterior CC) [32], no structural etiology, no background disorders, and patients aged < 6 years [70]) were reported to be favorable prognostic factors. In a study involving patients with West syndrome who presented with ES or tonic seizures, no developmental delay prior to the onset of epilepsy was found to be a favorable prognostic factor [6].

In terms of ES outcomes, Kanai et al. visually analyzed ictal polyphasic slow waves and reported that the symmetries of negative peaks, amplitudes, and durations were favorable prognostic factors [10]. Baba et al. analyzed presurgical recorded interictal scalp EEGs and reported that less power and connectivity of high-gamma activities were associated with favorable seizure outcomes post-CC [8]. Oguri et al. undertook computed frequency analyses of ranged delta to gamma waves during the ictal periods in the same patient group, and reported that fewer phase lags in the delta, theta, and gamma bands predicted favorable seizure outcomes after CC [11].

## 8. Surgical Treatment Selection: Resection Surgery or Corpus Callosotomy First?

### 8.1. Resection Surgeries and Adaptations for Epileptic Spasms

Several retrospective studies involving large patient populations have investigated resection surgeries including tuberectomy, lesionectomy, lobar or multilobar resection, anterior or posterior disconnections, and total or subtotal hemispherotomy or hemispherectomy for intractable ES [16,17,18,19,20]. Seizure-free ratios have been reported to range from 61% to 83%. These outcome data were relatively favorable compared with studies evaluating the outcomes of CC. However, patient backgrounds differed between those who underwent resection surgery and those who underwent CC.

Resection surgery was initially reported by Horsley in 1886, in which three adult patients with focal onset seizures had localized epileptogenic lesions surgically removed at London’s National Hospital [74]. To be a suitable candidate for resection surgery, patients with focal epilepsy, that is, with semiological, neurophysiological, neuroimaging, and nuclear medicine imaging findings indicating focal or lateralized epileptogenic regions, are required [25,34,35,75,76,77]. This concept of suitable candidates for resection surgery has also been applied to patients with ES or West syndrome [16,17,18,19,20,64,78]. In previous studies involving resection surgery for patients with ES, most patients had epileptogenic lesions with a positive ratio on MRI ranging from 83% to 100% and comprised 94% of the total patients reported (Table 2). CC studies with countable data have shown that the ratios of patients with brain lesions ranged from 0% to 65%, comprising 17% of the reported patients (Table 1). These ratio differences indicate different surgical adaptations for each surgical strategy. Resection surgeries are usually performed in unilateral hemispheres in each patient, and lateralization signs/findings are crucial for adaptation. Lesions observed on MRI that are concordant with seizure laterality, the distribution of discharges on scalp EEG, or abnormal areas on nuclear scanning, are appropriate lesions for resection regions, as are epileptogenic lesions [25,34,75,76]. As a representative study, Erdemir et al. reported 70 patients for whom resection strategies were decided based on lesions observed on MRI without invasive EEG monitoring [19]. The seizure-free ratio was 60% in their study, which was similar to that in other resection studies using invasive monitoring [19]. EEG and nuclear medicine imaging are also used to predict outcomes. Concordance between areas with interictal discharges on scalp EEG and MRI lesions or hypometabolism areas on positron emission topography (PET) in presurgical evaluations led to favorable findings in relation to seizure outcomes [20]. PET has also been reported to help with surgical decision-making and with guiding the placement of subdural electrodes in non-lesional MRI cases [16]. From these studies, focal or lateralized signs can be considered good indicators of resection surgery for ES, with the consistency of epileptogenic findings among each modality indicating a favorable predictive factor.

Functional deficits after the resection of eloquent cortices should be avoided as much as possible in resection surgeries [25]. Although the eloquent cortices include anterior or posterior language areas, visual cortices, and the primary motor area among others, hemiparesis after the resection of primary motor cortex is the critical for the patients with ES, who usually have severe cognitive impairment. The patients presenting with ES often have large epileptogenic zones and require multilobar resection or hemispherotomy/hemispherectomy [16,17,18,19,20]. If the patients present early pathologic handedness or hemiparesis, the resection surgery is considered relatively acceptable for the patients. If the epileptogenic zone is spread widely in the unilateral hemisphere, but avoids primary motor cortex on intracranial EEG, subtotal hemispherectomy, which spares the primary sensorimotor cortex, is considered a good procedure for the treatment of ES [16,51].

### 8.2. Characteristics of Patients Who Undergo Corpus Callosotomy

In previous studies regarding CC, patients tended not to have localized or lateralized signs or findings. The largest study concerning CC surgery for patients with ES by Baba et al. included 56 patients without MRI lesions. They reported that 43% of patients were seizure-free and that 23% of patients had a ≥80% seizure reduction postoperatively [6]. CC has also been performed for patients with bilateral diffuse or multiple lesions in patients with polymicrogyria, cortical tubers, or general atrophy on brain MRI [3,7,9,70]. Criteria for CC surgery to treat ES have included patients with no lesion or equally bilateral epileptogenic lesions selected for CC surgery. Factors such as presurgical severe developmental delay [6] and symmetrical ictal slow waves [10] on presurgical EEG findings have been associated with favorable CC outcomes. Based on advanced analyses for presurgical EEG, less power and less connectivity of high-gamma activities on presurgical scalp EEG [8] recordings and lower phase lags in ictal slow and gamma waves among bilateral hemispheres [11] have been associated with favorable seizure outcomes.

Uda et al. analyzed presurgical scalp EEG recordings using phase amplitude coupling analysis in patients who did not have MRI lesions, and reported that most patients had undergone CC surgery for intractable ES [12]. They hypothesized intra- and inter-hemispheric modulations for the emergence of ES, and estimated that patients classified as pattern type 1 (with focal onset ES confirmed using EEG and nuclear medicine imaging prior to CC surgery) could achieve seizure freedom via resection surgery, and classified other types (types 2–4) with unknown or generalized onset ES using presurgical evaluations and initially received CC. Those classified as type 2 (CC disclosed following focal onset ES) could achieve seizure freedom with additional resection, those classified as type 3 (presenting with residual generalized onset ES after CC) were not suitable candidates for additional resection, and those classified as type 4 (with spasms that emerged dominantly through inter-hemispheric modulation) could achieve seizure freedom only by undergoing CC surgery.

### 8.3. Types of Epileptic Spasms in Terms of Cortical Excitations and the Role of Corpus Callosum

**Figure 1:**
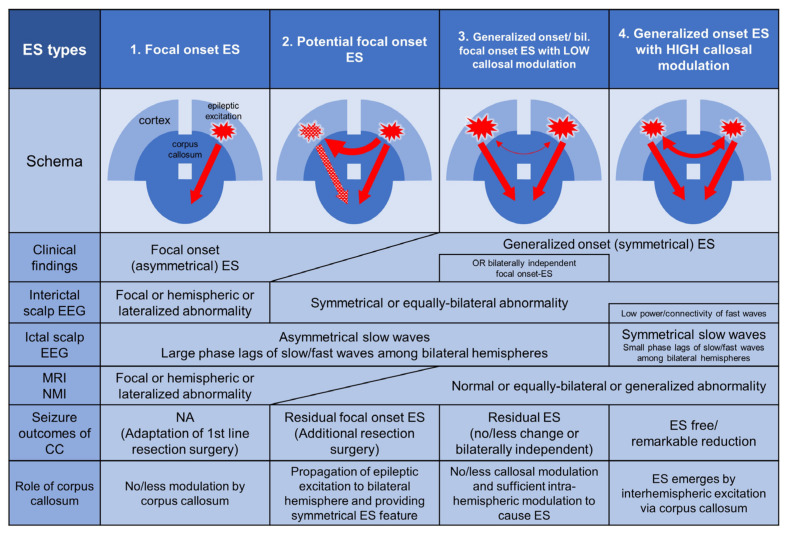
Four pattern types from the viewpoint of cortical excitation and the role of the corpus callosum and the characteristics, clinical features, neuroimaging, EEG, and surgical strategy. Type 1: Focal onset epileptic spasms (ES) are caused predominantly by unilateral cortices and tend to show a uniformly lateralized seizure type. These ES emerge with little or no contribution from the corpus callosum. Unilateral epileptogenic lesions on MRI or hypometabolic areas of nuclear medicine imaging are often detectable. The region or side of interictal EEG discharges tends to be concordant with lesions on brain imaging. Resection surgery can be performed based on presurgical evaluations. Type 2: Potential focal onset ES originates from the unilateral hemisphere; however, rapid bilateral propagation of epileptic excitations via the corpus callosum often emerge as generalized onset (symmetrical) ES. The epileptogenic hemisphere is relatively difficult to identify with presurgical evaluations, which were revealed to be not clearly localized/lateralized or had discordant localization/lateralization among the studies, and became apparent after corpus callosotomy (CC). Additional resection surgery is sometimes performed in the newly evident epileptogenic region. Type 3: Generalized onset ES or bilaterally independent focal onset (asymmetrical) ES with low callosal modulation can emerge predominantly through intrahemispheric modulations in each hemisphere. CC cannot prevent the emergence of ES. Type 4: Generalized onset ES with high callosal modulation occurs through predominant interhemispheric modulation via the corpus callosum, and CC can achieve seizure freedom without additional resection. Abbreviations: CC, corpus callosotomy; ES, epileptic spasms; MRI, magnetic resonance imaging; NMI, nuclear medicine imaging; NA, not applicable.

In Figure 1, we present four pattern types in relation to cortical excitation and the role of the corpus callosum, and we describe the characteristics of the role of the corpus callosum, clinical features, neuroimaging, EEG, and surgical strategy based on previous knowledge and experience. Focal onset ES (type 1) is caused predominantly by unilateral cortices and tends to show a uniformly lateralized seizure type. These emerge with little or no contribution from the corpus callosum. Unilateral epileptogenic lesions on MRI or hypometabolic areas of nuclear medicine imaging are often detectable. The region or side involved in interictal EEG discharges tends to be concordant with lesions on brain imaging. Resection surgery can be performed based on presurgical evaluations. Potential focal onset ES (type 2) originates from the unilateral hemisphere; however, it is clinically not lateralized due to the rapid propagation of epileptic activity via the corpus callosum. The epileptogenic hemisphere is relatively difficult to identify using presurgical evaluations, which do not clearly show localized/lateralized or discordant localization/lateralization among the studies, and which only became apparent after CC. Additional resection surgery is sometimes performed in the newly evident epileptogenic region. There are two types of generalized onset ES in which epileptogenic cortices are equally distributed in bilateral hemispheres. However, the role of the corpus callosum in the emergence of ES differs between these types. In generalized onset ES or bilaterally independent focal onset (asymmetrical) ES with low callosal modulation (type 3), intrahemispheric modulations in each hemisphere predominates in the emergence of the ES. CC cannot prevent the emergence of ES. However, in generalized onset ES with high callosal modulation (type 4), interhemispheric modulation among the bilateral hemispheres via the corpus callosum predominates in the emergence of the ES, and CC can achieve seizure freedom without additional resection. In these cases, ictal EEG is visually bilateral and synchronous on slow waves [10]. Computed frequency analyses of presurgical scalp EEG recordings have shown small phase lags of ictal slow and beta waves among the bilateral hemispheres and less power and connectivity on interictal records [8,11].

### 8.4. Surgical Selection Flow Proposals

**Figure 2:**
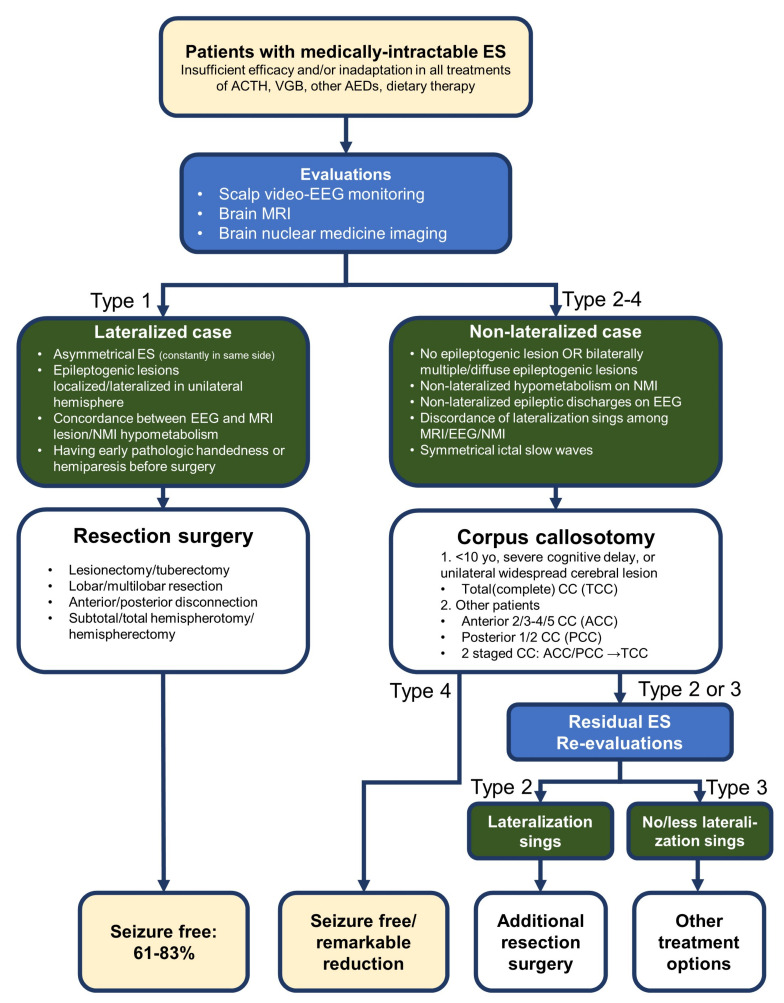
Selection flow of surgery for medically-intractable epileptic spasms (ES). If the epileptogenic region is identified in the unilateral hemisphere on the basis of lateralization signs—asymmetrical ES, epileptogenic lesion localized or lateralized in the unilateral hemisphere, concordance between localization of interictal discharges on EEG and MRI lesions, and concordance between localization of interictal discharges on EEG and hypometabolism in nuclear medicine imaging—the patient is presumed as lateralized case (ES type 1 in Figure 1) and resection surgeries (including frontal/posterior disconnection, subtotal or total hemispherotomy/hemispherectomy) are selected. Patients with early pathologic handedness or hemiparesis before surgery are considered relatively good candidates for resection surgery. If the patients have no, less or discordant lateralization signs—no or bilaterally multiple epileptogenic lesions, non-lateralized epileptogenic findings on EEG or nuclear medicine imaging, symmetrical ictal slow waves and/or discordance among findings of EEG/MRI and nuclear medicine imaging—the patient is presumed as non-lateralized case (ES type 2–4 in Figure 1) and corpus callosotomy (CC) is selected first. The ratio of seizure freedom in CC is estimated at 25–79% from previous studies (Table 1). If the patient is under 10 years of age, presenting severe cognitive impairment or unilateral widespread cerebral lesion, total CC is selected. In other patients, anterior, or posterior is initially recommended (the patients with unilateral widespread cerebral lesion can be considered for hemispherotomy/hemispherectomy). Additional total CC (two-staged CC) is considered in the patients with residual ES. Type 4 ES mostly ceases only by CC. If re-evaluations (mainly neurophysiological examinations) show lateralization signs, the ES is thought of as type 2 and additional resection surgery is recommended (seizure freedom: 43–71%). If the ES remains after CC (no change, bilaterally independent or milder ES than before), other treatments including antiepileptic drugs, dietary therapy and/or vagus nerve stimulations are selected.

Proposals for flow of surgical selection (resection surgery or CC) in patients with intractable ES are shown in Figure 2. In the schema, those patients with ES whose treatment had failed or who were not suitable for treatment with ACTH, vigabatrin, or other antiepileptic drugs, were evaluated using scalp video-EEG monitoring, brain MRI, and brain nuclear medicine imaging. If the epileptogenic region is localized in the unilateral hemisphere—which has been confirmed comprehensively using the following lateralization signs: asymmetrical ES (consistently in the same side), an epileptogenic lesion within the unilateral hemisphere, concordance of localization of interictal discharges on EEG and MRI lesions, and concordance of localization of interictal discharges on EEG and hypometabolism in nuclear medicine imaging—then resection surgery should be selected. If patients have no or fewer lateralization signs, that is, no or bilateral multiple epileptogenic lesions, non-lateralized epileptogenic findings on EEG or nuclear medicine imaging, symmetrical ictal slow waves and/or discordance of these signs, then CC is selected as first-line treatment. Patients who continue to have ES can receive additional resection surgery if re-evaluations (mainly neurophysiological examinations) show lateralization signs. If additional resection surgery is not appropriate, treatments using vagus nerve stimulation, dietary therapy, and other antiepileptic drugs can be attempted.

This selection flow may contribute to appropriate surgical treatments for patients with medically intractable ES. If patients do not show lateralization signs based on presurgical evaluations, CC can achieve seizure-free or sufficient seizure reduction, or disclose the latent epileptogenic region postoperatively in some patients.

## 9. Conclusions

CC has been a surgical treatment option for patients with medically intractable epilepsy for >80 years. Since 1993, CC has been applied for the treatment of drop attacks; however, the efficacy of CC in treating patients with ES remains unclear. Although some studies have reported increasing efficacy rates of CC for patients with ES, resection surgery studies have mostly involved the surgical treatment of patients with intractable ES. In those studies, patient backgrounds differed between those who had undergone resection surgery and those who had undergone CC. Based on the lateralized signs of clinical features, neuroimaging, and EEG in presurgical evaluations, we proposed a surgical selection flow involving resection surgery or CC as first-line treatment for patients with ES who had been assessed as suitable candidates for surgery. However, ES can persist for some patients who undergo CC who present with new lateralization signs, and additional resection surgery may be required in such cases.

## Figures and Tables

**Table 1:** Previous studies of corpus callosotomy (CC) for epileptic spasms (ES).

Authors		N: CC for ES (Total N of Any Size)	Age at 1st CC	Seizure Types (ES and Others)	MRI Lesion (+/-)	Procedures of Resection
1	Pinard et al. 1999 [62]	14 (17)	1.7–14.3 years	ES only: 36% ES + generalized: 64%	Unknown	ACC only: 2 ACC→TCC: 10 PCC→TCC: 2
2	Ono et al. 2011 [3]	7 (19)	0.4–3 years	ES only: 86% ES + focal + generalized: 14%	MRI lesion (+): 28% Bilateral: 28%	ACC→CR: 14% ACC→TCC→CR: 14% TCC→CR: 57% TCC→PQD: 14%
3	Otsuki et al. 2016 [4]	10 (30)	Unknown	Unknown	Unknown	TCC only: 100%
4	Iwasaki et al. 2016 [71]	8 (26)	1–14 years	ES only: 50% ES + generalized: 50%	MRI lesion (+): 50% Unilateral: 13% Bilateral: 38%	TCC only: 100%
5	Baba et al. 2018 [6]	56	0.4–1.9 years	ES only: 84% ES + generalized: 16%	MRI lesion (+): 0%	ACC only: 4% ACC→TCC: 5% TCC only: 91%
6	Baba et al. 2019 [8]	42	0.6–7 years	ES only: 43% ES + focal: 7% ES + generalized: 50%	MRI lesion (+): 21%	TCC only: 79% TCC→AQD/PQD/SHR/HR: 21%
7	Okanishi et al. 2019 [9]	7	2.1–21.5 years	ES only: 29% ES + focal: 29% ES + generalized: 43%	MRI lesion (+): 100% (bilateral tubers only)	TCC only: 71% TCC + CR/PQD: 29%
8	Kanai et al. 2019 [10] Oguri et al. 2020 [11] (Using the same patient data)	17	1.4–19.8 years	ES only: 18% ES + others: 82%	MRI lesion (+): 65%	ACC only: 18% TCC only: 82%
9	Uda et al. 2021 [12]	8 (10)	0.8–9.1 years	Unknown	MRI lesion (+): 0%	TCC only: 63% (5/8) TCC→AQD/PQD/HR: 37% (3/8)
**ES outcomes**			**Good prognostic factor**		**Others**	
1	Seizure free: 79% (after final CC)		NA		-	
2	Seizure free: 71% (TCC + others)		NA		This study reported only cases involving additional resection/disconnection.	
3	Seizure free: 70%		NA		Described as part of 67 surgical study cases	
4	Seizure free: 38%		MRI lesion (-)		-	
5	Seizure free: 43% > 80% reduction: 23%		low DQ		This study included only non-lesional cases	
6	Seizure free: 26% (after TCC) Seizure free: 43% (TCC ± others)		Low gamma power and connectivity (scalp EEG)		-	
7	Seizure free: 43% (after TCC) Seizure free: 71% (TCC ± others)		NA		Involved only patients with TSC. One patient achieved seizure freedom after taking everolimus additionally	
8	Seizure free: 41%		Symmetrical ictal slow waves (scalp EEG) Low interhemispheric phase lags (scalp EEG)		Some epileptic spasms were described as tonic spasms in Kanai et al.’s study [7]	
9	Seizure free: 25% (after TCC) Seizure free: 63% (TCC ± others)		NA		AQD without CC was selected for two patients based on a presurgical evaluation	

Abbreviations: ACC/PCC/TCC, anterior/posterior/total (complete) CC; AQD/PQD, anterior/posterior quadrant disconnection; CC, corpus callosotomy; CR, cortical resection; ES, epileptic spasms; HR, hemispherotomy/hemispherectomy; MRI, magnetic resonance imaging; NA, not analyzed; DQ, developmental quotient; SHR, subtotal HR; TCC, total corpus callosotomy; TSC, tuberous sclerosis complex.

**Table 2:** Previous studies of resection surgery for epileptic spasms.

Authors		N	Age at Surgery	Seizure Types or Symmetry of ES	MRI Lesion	Procedures of Resection
1	Chugani et al. 2015 [16]	65	5.1 ± 4.4 years (0.2–19 years)	ES only: 23% ES + focal: 58% ES + generalized: 11%	Lesion (+): 92%	Hemispherectomy: 31% Subtotal hemispherotomy: 26% Multilobar: 20% Lobar ± tuberectomy: 14% Tuberectomy: 9%
2	Barba et al. 2016 [17]	80	5.8 ± 4.0 years	Symmetric ES: 49% Asymmetric ES: 24% Both types: 28%	Lesion (+): 96%	Lobectomy: 72.5% Multilobar/hemispherotomy: 27.5%
3	Chipaux et al. 2017 [18]	59	4.6 ± 3.5 years (0.3–16 years)	ES only: 15% ES + others: 85%	Lesion (+): 96%	Hemispherotomy: 34% Lesionectomy: 8% Lesionectomy + cortical resection *: 46% Frontal disconnection: 8% Posterior disconnection: 3% Endoscopic HH removal: 3%
4	Erdemir et al. 2021 [19]	70	1.9 ± 1.6 years	ES only: 46% ES + others: 54% Symmetric: 54% Asymmetric: 40% Unknown symmetry: 6%	Lesion (+): 100% Bilateral: 24% Unilateral: 76%	Hemispherectomy: 44% Lobectomy/lesionectomy: 33% Multilobar resection: 23%
5	Liu et al. 2021 [20]	64	3.3 ± 2.7 years	ES only: 64% ES + others: 36%	Lesion (+): 83%	Hemispherotomy/subtotal hemispherotomy: 27% Multilobar: 11% Lobar: 34% Tuberectomy: 23% Unknown: 3%
**Outcomes**			**Good Prognostic Factor**		**Others**	
1	Seizure free: 71%		Short epilepsy duration MRI lesion (+)		22 patients discontinued medication after surgery	
2	Seizure free: 61%		Complete resection of SOZ MRI lesion (+) Low age at surgery/short epilepsy duration		ECoG monitoring: 30%	
3	Seizure free: 75%		Low age at surgery		Visible epileptogenic lesions on MRI in most cases. Eight patients were declined surgery following presurgical evaluation because of multiple foci.	
4	Seizure free: 60% Improvement: 24%		Lobar/sublobar epileptogenic lesion		MRI-oriented surgery No invasive EEG (ECoG)	
5	Seizure free: 83% **		Concordances: between MRI and scalp EEG between PET and scalp EEG Frequent interictal gamma activities (scalp EEG) Intraoperative continuous discharges (ECoG)		ECoG monitoring: 95% Stereo-EEG: 20%	

Abbreviations: ES, epileptic spasms; HH, hypothalamic hamartoma; SOZ, seizure onset zone; MRI, magnetic resonance imaging; PET, positron emission tomography; ECoG, electrocorticography; EEG, electroencephalogram. * Resection of cortex with seizure onset zone in ECoG array; ** residual aura was accepted.

## Data Availability

This research does not include any data which needs availability statement.
